# Facile preparation of flame-retardant cellulose composite with biodegradable and water resistant properties for electronic device applications

**DOI:** 10.1038/s41598-023-30078-0

**Published:** 2023-02-23

**Authors:** Saravanan Chandrasekaran, Alvaro Cruz-Izquierdo, Remi Castaing, Baljinder Kandola, Janet L. Scott

**Affiliations:** 1 Centre for Sustainable Chemical Technologies and Department of Chemistry, University of Bath, Claverton Down, Bath, BA2 7AY UK; 2 Material and Chemical Characterisation Facility (MC2), University of Bath, Claverton Down, Bath, BA2 7AY UK; 3 Institute for Materials Research and Innovation, University of Bolton, Deane Road, Bolton, BL3 5AB UK; 4 Department of Chemistry, School of Engineering, Presidency University, Rajanukunte, Itgalpura, Bangalore, 560064 India

**Keywords:** Chemistry, Materials science

## Abstract

The aim of the present study is to produce flexible, flame-retardant, water-resistant and biodegradable composite materials. The ultimate goal of this research is to develop simple processes for the production of bio-based materials capable of replacing non-degradable substrates in printed circuit board. Cellulose was chosen as a renewable resource, and dissolved in 1-ethyl-3-methylimidazolium acetate ionic liquid to prepare a cellulosic continuous film. Since flame retardancy is an important criterion for electronic device applications and cellulose is naturally flammable, we incorporated ammonium polyphosphate (APP) as a flame-retardant filler to increase the flame retardancy of the produced materials. The developed material achieved a UL-94 HB rating in the flammability test, while the cellulose sample without APP failed the test. Two hydrophobic agents, ethyl 2-cyanoacrylate and trichloro(octadecyl)silane were applied by a simple dip-coating technique to impart hydrophobicity to the cellulose-APP composites. Dynamic mechanical analysis indicated that the mechanical properties of the cellulosic materials were not significantly affected by the addition of APP or the hydrophobic agents. Moreover, the biodegradability of the cellulosic materials containing APP increased owing to the presence of the cellulase enzyme. The hydrophobic coating slightly decreased the biodegradability of cellulose-APP, but it was still higher than that of pure cellulose film.

## Introduction

Electronic products such as laptops, smartphones, televisions, tablets are pervasive in our everyday life and their usage is increasing with time. The e-waste (electronic and electrical waste) from the used devices is one of the fastest growing unavoidable waste streams globally^1^. According to the United Nations environment program report, we are producing 50 million tonnes of e-waste every year. Only less than 20% of e-waste is formally recycled, while the remaining e-waste ends up in landfill or is informally recycled^2^. Informal recycling process not only leads to health and pollution impacts, but also creates significant loss of scarce and valuable raw materials. For example, one tonne of e-waste contains approximately 100 times more gold than one tonne of gold ore^2^. Therefore, it is important to recover the valuable metals from e-waste.

Green electronics is an emerging field of research that aims to manufacture devices in a sustainable and environmentally friendly way from naturally occurring non-toxic materials. Biodegradable electronics is a part of green electronics where materials are biodegradable, renewable and compostable, with minimum amount of toxic materials.The main functional unit in an electronic device is the printed circuit board (PCB), made of metals (mainly copper, lead, tin, aluminium, gold, silver and platinum), non-metals (organic materials), ceramic (aluminium materials) and polymeric materials^3^. Therefore, the increase in demand of electronic products results in an increase in demand of petrochemical raw materials (for organic and polymer materials) and valuable metals from earth. Generally, petroleum-based polymeric materials are non-biodegradable and create complications in the recovery process of metals from the used electronic products^4,5^. Therefore, using biodegradable PCBs will help recovering the valuable metals, separate the toxic materials from e-waste, and also will address their environmental and economic impacts.

Cellulose has been widely used in many fields such as pharmaceutical industries, biofuel production, adsorbents, paper transistors, biosensors, energy storage devices, 3D printing, wearable electronics, electromagnetic interference shielding, piezoelectric nanogenerators (PENGs), triboelectric nanogenerators (TENGs) and hybrid piezo/triboelectric nanogenerators (PTENGs) for energy harvesting and other intelligent electronics sectors^6–20^ and can also be used to replace the non-biodegradable polymers in electronic circuit boards^21,22^. This is because cellulose has excellent mechanical properties, is the most abundant biomass material available in nature, is renewable and non-petroleum sourced, shows biocompatibility, biodegradability, and surfaces can be easily functionalised chemically^23,24^. However, the flammability and insolubility associated with non-processability of cellulose restrict its application in various fields. Therefore, it is very important to find a suitable solvent medium to convert the powder form of cellulose into a film form, while reducing the flammability of the cellulosic material. Recently, ionic liquids (ILs) have been explored as environmentally friendly and reusable solvents to dissolve the cellulose^25–32^. Moreover, various halogenated and non-halogenated materials can be used to improve the flame retardancy of cellulose^33,34,35–42^. From various commonly used FR materials (e.g., non-halogenated materials, metal oxides, and inorganic salts), ammonium polyphosphate (APP) has been chosen owing to its low cost, high phosphorous and nitrogen contents, efficient FR efficiency even when present in small quantities and environmental friendliness associated with its condensed phase action^43–45^.

On the other hand, the prepared cellulose-APP composites for electronic device (e.g., PCB) applications, the surface of the composite material must be suitable for the conductive ink printing process. The wettability, hydrophilicity and hydrophobicity of the materials surface have a great influence on printing performance^46,47^. Therefore, it is necessary to find the water absorption or resistance property of the composite materials. As cellulose contains several hydroxyl groups in the structure, cellulose is hydrophilic in nature and tends to absorb moisture. Due to the above reason, surface modification from hydrophilic to hydrophobic is required to enhance the water-resistant property and find the suitability of the cellulose composite for printing process. The hydrophilicity of cellulose can be reduced by using hydrophobic agents which can further broaden their utilisation in various fields^48–50^.

In this work, we have prepared a cellulose-APP composite by dissolving cellulose (and filler) with recoverable and reusable 1-ethyl-3-methylimidazolium acetate (EMIMAc) as solvent medium. Two hydrophobic agents were utilised to improve the water resistant property to the cellulose films. Then, the prepared composites were subjected to thermal, flammability, water resistibility, mechanical and biodegradation studies to verify the suitability of the cellulose-APP composites for biodegradable PCB application.

## Materials and methods

### Experimental details

#### Materials

α-cellulose (Sigma) was dried under vacuum (rota-vapor) for 4 h at 80 °C, 1-ethyl-3-methylimidazolium acetate (EMIMAc) (BASF, Basionics, > 95wt%) was kept under vacuum (Schlenk line) for 4 h at 90 °C. Calcium carbonate (Aldrich) and aluminium oxide (Riedel–de Haen) were kept under vacuum for 6 h at 110 °C to remove the moisture from metal oxides. Ammonium polyphosphate (Chemox Pound, n > 1000) was used as received. HPLC grade methanol, diethyl ether, toluene, ethyl 2-cyanoacrylate (E2CA), trichloro(octadecyl)silane (TOS) from Aldrich and ethanol (VWR Chemicals) were used as received. Dried toluene was used to dissolve the trichloro(octadecyl)silane. Celluclast 1.5 L produced by *Tricoderma reesei* ATCC 26921, citric acid monohydrate, 3,5-dinitrosalicylic acid (DNS), sodium hydroxide, sodium potassium tartrate, phenol and sodium metabisulphite were obtained from Sigma-Aldrich. Bake-o-glide non-stick baking sheets (polytetrafluoroethylene (PTFE)-coated fabric) were purchased from the local vendor.

#### Preparation of cellulose-APP composite film

Brabender plastograph was used as a mixer to prepare the cellulose-APP composites in the presence of EMIMAc as the dissolution medium. To prepare the composite, we used a slightly modified method of our previously reported method^46,50^. The following modifications were made to the preparation method: we sonicated the APP in EMIMAc and increased the reaction time to 35 min. In our previous work 10 wt% of FRs (with cellulose) was showing good flame-retardancy and mechanical property^46,50^. Therefore, we set the APP weight ratio at 10 wt% relative to the total weight of cellulose and APP used in the dissolution process. The 10 wt% APP cellulose composite was prepared as follows: 1.3 g of APP was dispersed in 68 g of EMIMAc (85 wt% w.r.t the total weight of cellulose and EMIMAc) and sonicated for 15 min to disperse APP in EMIMAc. The mixer was pre-heated to 90 °C and the blade rotation speed was set to 70 rpm. Then dispersed APP in EMIMAc solution was added into the pre-heated mixer and allowed to rotate for 10 min. 12 g of α-cellulose (15 wt% w.r.t the total weight of cellulose and EMIMAc) was divided into 4 × 3 g and added into the mixer consecutively for every 5-min. Finally, the mixing was allowed for another 10 min at 90 °C. The total mixing time was 35 min. The resultant composite was recovered from the mixer and kept in an air-tight container.

Cellulose-APP composite films were prepared in the hydraulic press equipped with the heating unit (Moore, UK). 14 g of above prepared cellulose-APP composite material was kept in-between non-stick bake-o-glide sheets and pressed into a film with the hydraulic press. A uniform pressure was maintained at 80 psi at 90 °C for 30 min. At the end, cellulose film casted on bake-o-glide was removed and subjected to the regeneration process^50^. The cellulose-APP-EMIMAc mixture within the bake-o-glide film was placed in a methanol bath for 15 min. In this way, EMIMAc will be dissolved into methanol while cellulose is regenerated into a continuous film form. The bake-o-glide layers were slowly peeled-off to recover the final composite. The obtained film was subjected to further methanol washes to completely remove any EMIMAc left in the cellulose film. Finally, the regenerated film was dried at room temperature (RT) using embroidery hoop as a support to obtain a non-curly film^50^. EMIMAc in methanol solvent was recovered by removing the methanol in the rotary evaporator.

### Surface modification of cellulose-APP composite film via hydrophobic agents’ treatments

We used a previously established and reported method with minor modifications to perform surface modification of cellulose-APP composite using TOS and E2CA^50^. The amount of the hydrophobic agents used was 10 wt% with respect to the total mass of the composite film. Here, we have adopted a simple dip-coating technique to coat the composite with hydrophobic agents.

1. Trichloro(octadecyl)silane (TOS) on cellulose-APP composite

The procedure for the preparation of hydrophobised cellulose composites involved coating the hydrophobic agent on the surface of cellulose composites by immersing the film into 23 mg (0.003 M) of TOS in 19.8 g of dry toluene solution for 30 min at room temperature. The whole assembly was kept under inert (nitrogen) atmosphere. Afterwards, the cellulose composite film was removed and dried at room temperature for 10 min. The same procedure was repeated to make sure that the composite surface was completely coated with TOS. After drying at RT for 10 min, the coated cellulose composite was washed with diethyl ether (20 ml) twice to remove any acidic residues. The resultant modified film was dried at RT overnight and subjected to characterization studies. The procedure for TOS coating in ethanol (EtOH) was same as above except that ethanol was used instead of toluene.

(B) Ethyl 2-cyanoacrylate (E2CA) on cellulose-APP composite

Since E2CA reacts with glass, the mixture was prepared in a plastic vessel at 0.003 M (43 mg in 114.5 g of toluene) and the dip-coating was performed at 4 °C. The whole assembly was kept under inert nitrogen atmosphere. Afterwards, the cellulose composite film was removed and dried at room temperature for 10 min. The same procedure was repeated to make sure the composite surface was completely coated with E2CA. The resultant surface modified composite film was dried at RT for overnight and subjected to characterization studies.

### Characterization techniques

The thermal oxidative degradation stability of the regenerated cellulose and their composites with inorganic material was analysed by thermogravimetric analysis (SETARAM instruments, TGA 92) under air atmosphere at the heating rate of 5 °C/min. Each sample was kept at isothermal condition (100 °C) for 30 min in air atmosphere to eliminate the moisture from the cellulose composites and TGA was performed in a desired temperature range from 25 to 400 °C. 400 °C as the maximum temperature was chosen because up to this temperature decomposition of cellulose is complete and above this oxidation of char occurs^33,51^.

Flammability tests UL-94 HB (horizontal) and LOI were carried out at the University of Bolton, UK. Samples (composites) for flammability studies were cut into rectangular shape (125 mm × 13 mm) with the thickness range from 0.06 to 0.07 mm. Each cellulose composite films were marked with two lines perpendicular to the longitudinal axis of the bar, 25 ± 1 mm and 100 ± 1 mm from the end that is to be ignited and they were subjected into the horizontal flame test in accordance with ASTM D 635-03 standard method. The sample was clamped horizontally and ignited with a burner. The flame spread was calculated from the time required to burn the defined distance. The LOI measurements were carried out with Fire Testing Technology, UK according to ASTM D 2863 standard method. The sample was held vertically in the glass chamber of the instrument in which the flow of oxygen and nitrogen mixture gas was controlled. The LOI values of the composites were calculated from the required oxygen concentration in the mixture of gases for sustained burning for requited length. All the measurements were repeated three times and the results averaged. Attenuated total Reflectance-Fourier transform infrared spectroscopy (PerkinElmer, ATR-FTIR) was recorded in the frequency range from of 4000 to 550 cm^−1^.The surface morphology of hydrophobic agent treated and untreated cellulose-FR composite was studied using a scanning electron microscope (JEOL JSM-6480).

Contact angle (CA) measurements were made using the sessile droplet method in air at room temperature with 2 µL droplets of deionized (DI) water as the solvent. The cellulose composite was fixed on the glass plate with the support of cello tape and placed on the goniometer and the contact angle was determined 10 s after the water droplet was deposited on the surface of the cellulose composites. Three samples were used and each data point was an average of 5 measurements on each sample. The time dependent contact angle values were measurements in all the non-hydrophobised and hydrophobised sample after 10 s, 5 min and 15 min after water droplets were deposited on the surface of the cellulose composites.

A Setsys Evolution TGA 16/18 from Setaram was used for thermogravimetry coupled with evolved gas analysis; the Calisto program was employed to collect and process the data. 18–20 mg of cellulose film was loaded into an alumina crucible and TGA was performed in the temperature range of 30–400 °C under argon atmosphere at a heating rate of 5 °C/min (after an argon purge for 40 min). During the heating ramp, evolving gases were transferred from the analytical chamber to a mass spectrometer through a stainless-steel capillary. The mass spectrometer was an Omnistar GSD 320 by Pfeiffer Vacuum, equipped with a quadrupole mass analyser and a Faraday detector.

Mechanical properties were measured using a dynamic mechanical analyser DMA1 from Mettler Toledo, with the data acquisition program STARe. Rectangular specimens of 25 mm × 10 mm × 0.06–0.07 mm were clamped in the tension clamping assembly leaving a free length of 10 mm; the cross-section area was then 0.60–0.70 mm^2^. The elastic moduli and damping factor were measured at a strain amplitude of 0.1% at a frequency of 1 Hz. The testing temperature was fixed to 30 °C using circulated water, and the humidity was set to 20% RH or 70% RH using a modular humidity generator proUmid MHG32; these two humidity values correspond to the low end and high end values of RH on earth reported in the literature^52^. The elastic modulus was measured after 60 min of soaking at the given humidity value, to allow enough time to reach a stable damping factor.

### Biodegradability studies

Celluclast 1.5L was used to degrade the films. The cellulase activity was determined by measuring soluble reducing sugars via DNS assay^53^. A typical enzymatic hydrolysis consisted of 2.5 mg of sample, 0.2 mL of sodium citrate buffer (50 mM, pH 5.0), and Celluclast 1.5 L (1 Filter Paper Unit/g of sample) and kept for 1 h at 50 °C. All reactions were run in triplicate.

### Ethical approval

This article does not contain any studies with human participants or animals performed by any of the authors.

### Consent to participate

All authors have participated in the writing of the manuscript and given their consent to submit the manuscript.

## Results and discussion

### Preparation of cellulose-APP composite

The prepared cellulose-APP composite film with thickness around 0.06–0.07 mm was subjected to visual inspection and found that cellulose-APP film was transparent which indicates a uniform dispersion of APP in the cellulose matrix, as also seen by scanning electron microscope (SEM) images, discussed in a later section.

### Characterization of cellulose-APP composite

#### Effect of APP on thermal and flame retardant properties of cellulose-APP composite

**Figure 1:**
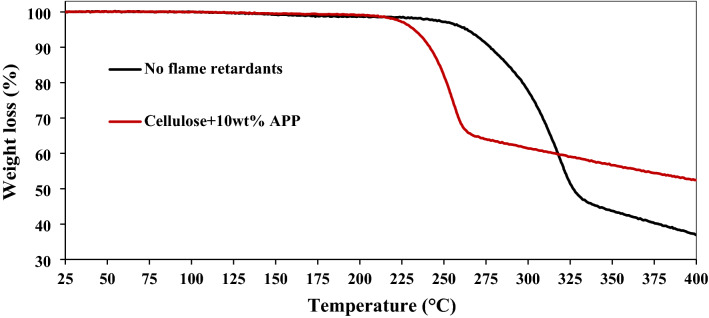
TGA analysis on cellulose-APP composites with 10 wt% APP under air at 5 °C/min.

The thermal stability of the prepared cellulose-APP composite was analysed by thermogravimetric analysis (TGA) under air atmosphere. The onset of degradation temperature (T_Onset,_ temperature at which mass loss starts) of cellulose is ~ 260 °C^54^. The addition of 10 wt% APP reduced the thermal stability of the cellulose film, with a reduction in T_Onset_ from 260 to 225 °C and more mass loss than cellulose in the temperature range 225–315 °C (Fig. 1). This can be related to the decomposition of APP into phosphoric acid, the latter then catalyses the dehydration of cellulose, leading to less laevoglucosan and more char formation as discussed in detail by Kandola et al.^33^. The higher charring tendency of cellulose-APP can be seen from reduced mass loss above 315 °C (increased thermal stability). The increased char formation is indication of lower flammability of the composites^55,56^.

The flame retardant properties of cellulose without and with flame retardant were evaluated in terms of LOI and UL-94 HB rating (Table 1). The LOI value of the cellulose composites increased from 19 to 25.3% when 10 wt% APP was present (Table 1). Moreover, the UL-94 HB test results indicating that 10 wt% APP provides a self-extinguishing fire character to the cellulose (Table 1). This could be explained as, when cellulose composite subjected into ignition, as mentioned earlier, the APP undergo thermal degradation producing phosphoric acid and ammonia^33^. The produced phosphoric acid effectively promotes the dehydration of the hydroxyl groups of cellulose as opposed to laevoglucosan formation and hence, suppressing formation of combustible volatiles. The dehydrated cellulose undergoes crosslinking reactions leading to char formation^33^. In addition, APP on decomposition produces ammonia, a non-flammable gas, which also acts as a diluent for flammable gases. The non-flammable gas production and char formation as a condensed phase help in achieving UL-94 HB rating^57–59^. The above LOI and UL-94 HB results indicate that char produced from APP acts as a protective barrier and hence, APP works as an efficient intumescent flame-retardant on the cellulose matrix.

**Table 1:** Effect of addition of APP on the limited oxygen index and flammability of the cellulose.

Cellulose composites	LOI (%) (ASTM-D2863)	Flammability tests UL-94 HB, time to burn marked area of samples in secs (Std. dev) (ASTM D 635-03)	Test result (pass = burning rate is less than 3"/min)
No flame retardants	19	23.7 (± 1.2)	Fail
10 wt%—APP	25.3	Self-extinguished	Pass

Furthermore, the thermal and flame-retardant properties of cellulose have been investigated by incorporating various other inorganic compounds such as calcium carbonate (CaCO_3_) and aluminium oxide (Al_2_O_3_) as flame-retardants into cellulose (Fig. S-1 and Table-S-1). Among the various flame-retardants tested, APP was the most effective at increasing cellulose's flame retardancy. As a result, no further research on the CaCO_3_-cellulose and Al_2_O_3_-cellulose composites has been conducted.

### Surface functionalization of cellulose-APP composite

#### Preparation of hydrophobised cellulose-APP composite

**Figure 2:**
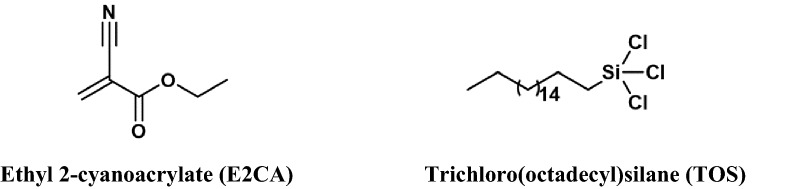
Structure of hydrophobic agents.

**Figure 3:**
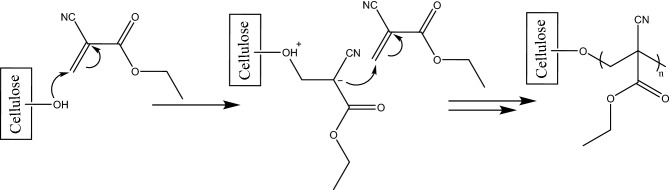
Plausible mechanism of addition of hydrophobic agent into the surface of cellulose film.

Hydrophobic cellulose composites were prepared by single-step, simple and cost effective method by treating with a relatively low concentration of the hydrophobic agents (0.003 M). In this work, we have chosen E2CA and TOS as the hydrophobic agents (Fig. 2) to introduce the hydrophobicity in the cellulose-APP composites and the films were dip-coated twice to ensure an adequate coverage of the hydrophobic agents. Figure 3 depicts the surface functionalization of cellulose as a representative reaction between a hydrophobic agent (E2CA) and cellulose. Since the concentration of the hydrophobic agents is relatively low, we were unable to measure the weight difference between untreated samples and samples after hydrophobic agent treatment. The quantity of hydrophobic agents added on cellulose-APP composite is very less. It is envisaged that the small concentration of the agents will not adversely affect the flammability. Therefore, flammability tests on the hydrophobised composite were not conducted. However, the resultant hydrophobised cellulose composites were subjected to further analysis to characterise the presence of E2CA and TOS.

#### Attenuated total reflectance-Fourier transform infrared spectroscopy (ATR-FTIR) of non-hydrophobised and hydrophobised cellulose-APP composite

**Figure 4:**
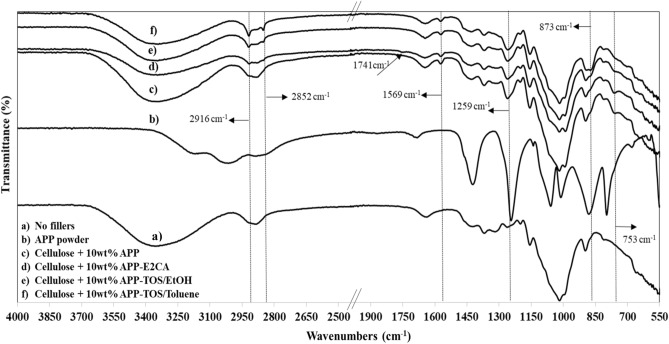
ATR-FTIR of (**a**) cellulose with no flame-retardants, (**b**) APP powder, (**c**) cellulose-APP composite, (**d**) cellulose-APP composite—E2CA coated, (**e**) cellulose-APP composite—TOS/EtOH coated and (**f**) cellulose-APP composite—TOS/Toluene coated.

The ATR-FTIR spectra of cellulose, APP-powder, non-hydrophobised and hydrophobised cellulose-APP composite with 10 wt% APP are shown in Fig. 4a–f. The bands at 3353 cm^−1^ and in the range from 2927 to 2879 cm^−1^ are hydroxyl group and –CH/–CH_2_ stretching vibration of cellulose respectively. The band at 1640 cm^−1^ is attributed to presence of water in the cellulose. A weak band at 1569 cm^−1^ may be due to charged –NH^+^ or COO^−^ from carbamic acid formation during dissolution of cellulose in the presence of APP, 1259 cm^−1^ (–P–O), 873 cm^−1^ (–P–O asym str vib) and 753 cm^–1^ (O=P–O) are attributed to the presence of APP in the cellulose composites (Fig. 4c). The bands at 2916 and 2852 cm^−1^ are –CH and –CH_2_ stretching vibration of alkyl chain of the hydrobhobising agents polyethyl 2-cyanoacrylate (PECA, the polymeric form of E2CA) and TOS (Fig. 4d–f). E2CA hydrophobised cellulose composite (Fig. 4d) shows an additional band at 1741 cm^−1^ that is related to –C=O stretching in the ester group of PECA^46,60^. The above ATR-FTIR analysis confirmed that both hydrophobic agents were deposited on the surface of the cellulose composites, which improved the water resistance of the cellulose composites (see “Results” below).

#### Water resistance characteristics of cellulose-APP composites

**Figure 5:**
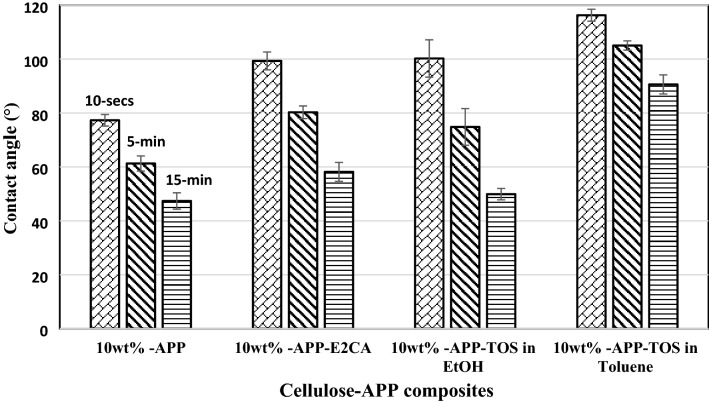
Contact angle measurements and water resistance property analysis on non-hydrophobised and hydrophobised cellulose-APP composite.

**Figure 6:**
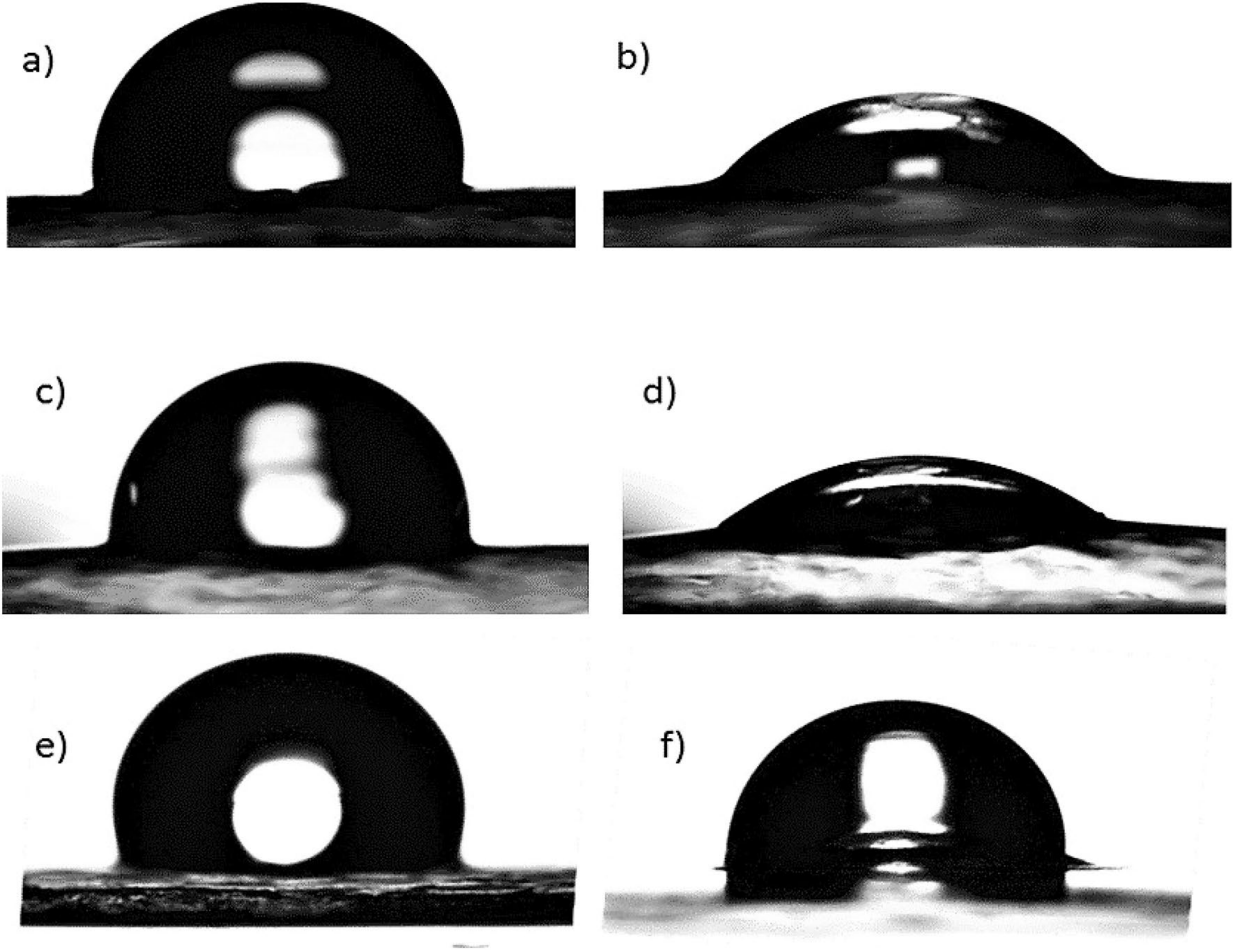
Representative images of surface contact angle measurements on cellulose-APP composites coated with (**a**) E2CA/10 s, (**b**) E2CA /15 min, (**c**) TOS-EtOH/10 s, (**d**) TOS-EtOH/15 min, (**e**) TOS-Toluene/10 s and (**f**) TOS-Toluene /15 min.

The water resistance properties of non-hydrophobised and hydrophobised cellulose-APP composites were evaluated by water contact angle measurements (Fig. 5). The as-prepared non-hydrophobised cellulose composite with 10 wt% APP showed a higher contact angle value 77° than cellulose without APP (CA = 50°, not included in the figure). This may be due to the fact that the surface roughness of the cellulose is slightly increased in presence of APP. However, the contact angle value is within the hydrophilic range (CA = 0°–90°). The CA increased from 77° to 99° when the cellulose-APP composite was coated with E2CA as a hydrophobic agent and the CA value is reduced from 99° to 80° and 58° while observing the water absorption nature of the cellulose-APP composite with time (water evaporation was not taken into account, Fig. 6). This may be due to the instability or hydrolysis of PECA and subsequent loss on surface functionality followed by absorption or penetration of water into the cellulose-APP composite. When the cellulose-APP composite was treated with TOS in ethanol solvent, there was not a remarkable increase in the contact angle value (CA = 100°) and the water resistance property remained similar as the composite coated with E2CA. However, the contact angle and the water resistivity of the composite was increased from 100° to 116° by varying the solvent from TOS in ethanol to TOS in toluene. The increase in hydrophobicity and the water resistance property of the composite by varying the solvents can be attributed to the fact that TOS may interact with hydroxyl group of ethanol and reduces the interaction between the TOS and cellulose leading to less amount of TOS deposition on cellulose. Besides, the cellulose film treated with TOS in toluene showed a good durability against water absorption with contact angle value > 90° even after 15 min of water droplet deposition on the surface of the cellulose-APP composite. These results demonstrate that the surface wettability of the materials can be controllable by altering the hydrophobic agents and the solvents used during the surface modification process. From the above studies, we believe that the water resistance property of hydrophobised cellulose-APP composite is as good as conventional PCB materials^61^ and the surface can be modifiable according to the requirement for the printing process.

#### Surface morphology of non-hydrophobised and hydrophobised cellulose-APP composites

**Figure 7:**
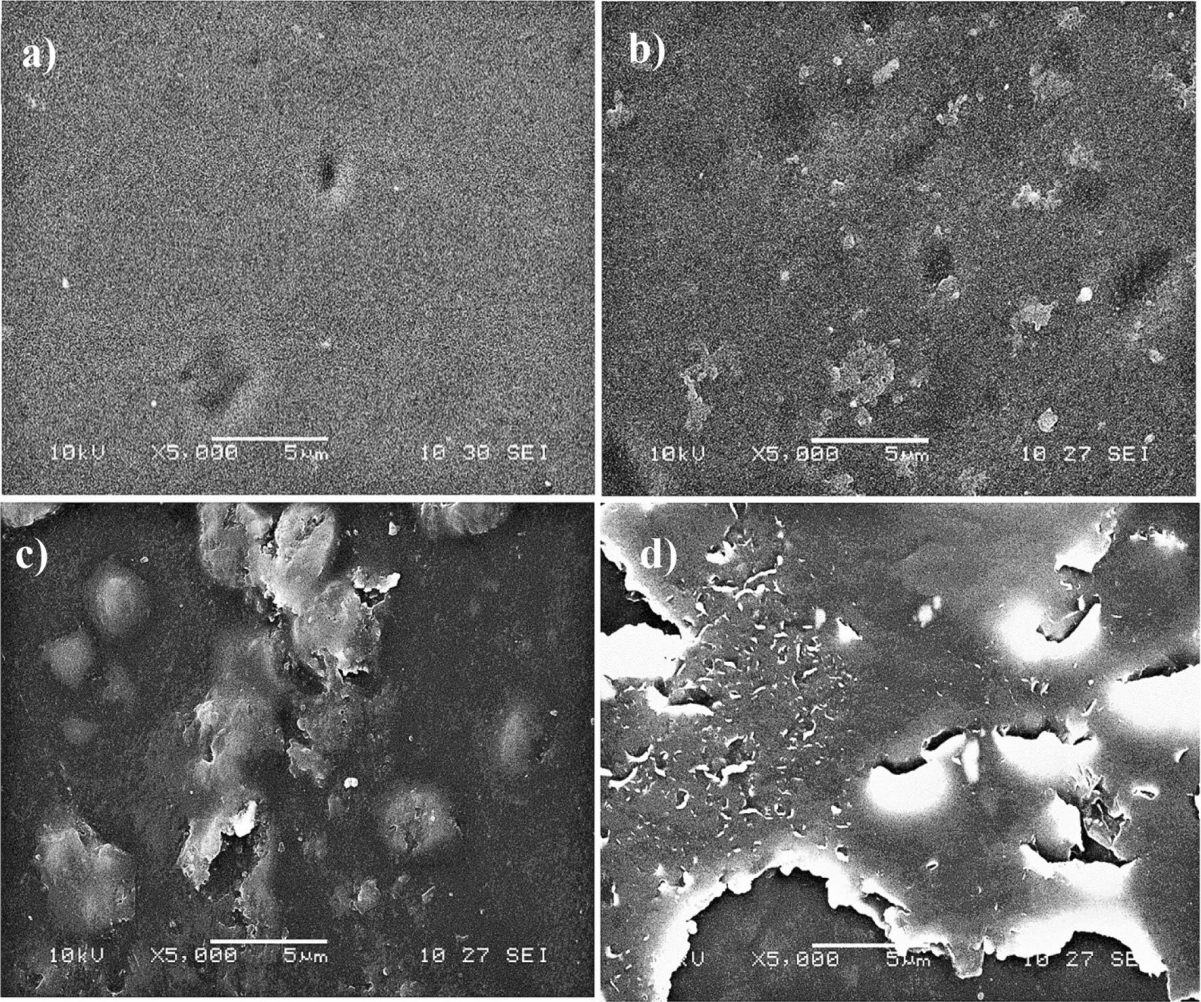
SEM pictures of hydrobhobising agents treated cellulose-APP composite (**a**) before surface treatment, after treatment with (**b**) E2CA, (**c**) TOS in ethanol and (**d**) TOS in toluene.

SEM technique was used to evaluate the effect of the hydrophobic agent on the surface of the cellulose-APP composite (Fig. 7). The surface morphology is more distinguishable with the nature of hydrophobic agents and the solvents used in the functionalisation reaction (Fig. 7a–d). Figure 7a reveals that the non-hydrophobised cellulose-APP composite has a smooth surface with absence of any solid layer on the surface. After hydrophobic agent treatment, additional layer formed on the surface of the cellulose-APP composite (Fig. 7b–d). As shown in Fig. 7b–d, layer deposition is higher with TOS treated composite (Fig. 7c) than E2CA treated composite (Fig. 7b) and further improvement was observed while changing the TOS solvent medium from ethanol to toluene (Fig. 7d). This may be due to the fact that the interaction between the cellulose-APP composite and hydrophobic agent is more favourable in presence of TOS in a nonpolar solvent (toluene). This is directly correlated to high water contact angle values with high water resistance property of cellulose-APP composite hydrophobised with TOS in toluene solvent.

#### Thermal stability of non-hydrophobised and hydrophobised cellulose-APP composites

The hydrophobised cellulose-APP composite were subjected to thermal degradation studies under air atmospheres. The hydrophobised cellulose-APP composites showed a similar degradation behaviour as non-hydrophobised cellulose-APP composites (Fig. S-2). This indicates that the surface functionalization has no significant influence on the thermal degradation of cellulose-APP composites. Carbon dioxide and water are the main by-products during the pyrolysis of cellulose. Therefore, evolving gas analysis was also carried out under inert atmosphere using TGA coupled with a mass spectrometer and monitored the evolution of carbon dioxide and water vapour (see the supporting information, Fig. S-3), which showed no significant difference between the non-hydrophobised and hydrophobised samples.

#### Mechanical properties of cellulose-APP composite at high and low relative humidity (RH) atmosphere

The elastic modulus of cellulose-APP composite films was measured using a dynamic mechanical analyser at fixed temperature (30 °C) and two humidity conditions (20% or 70% RH) (Table 2). At a first glance, the elastic modulus is systematically lowered when the humidity increases: the value at 70% RH is half of the value at 20% RH, which indicates that moisture absorption increases its flexibility, which confirms the plasticizing effect on cellulose^62^ regardless its functionalization (or lack of it). The large sample variability prevents a clear interpretation of the effect of surface treatment, therefore analysis of variance (ANOVA) was used to calculate the difference in modulus given the standard variation in each group^63^. It was proven at a confidence level of 90% that the surface treatment had a significant effect on the modulus at both low and high humidity contents (0.049 and 0.075 *p* values, respectively). More precisely, it was proven that the composite hydrophobised with E2CA significantly increases the elastic modulus compared to the non-hydrophobised composite; no such difference could be proven with the other hydrophobic agent TOS. So, there is not a detrimental effect on the stiffness of those films. Besides, all cellulose-APP composites showed an elastic modulus of about 1 GPa or above, even at high humidity, which makes them appropriate for being used in PCB as a substrate material.

**Table 2:** The elastic modulus of cellulose-APP composites with various surface treatments at 20% and 70% RH; each condition was tested on five different specimens and the errors are given at 95% confidence.

Surface treatment	Elastic modulus at 20% RH (GPa)	Elastic modulus at 70% RH (GPa)
No treatment	1.9 ± 0.2	0.9 ± 0.1
E2CA	4.3 ± 2.3	1.8 ± 0.9
TOS in ethanol	2.2 ± 0.7	1.1 ± 0.3
TOS in toluene	3.1 ± 2.2	1.5 ± 1.0

#### Conductive Ag‑Ink printing studies on non-hydrophobised and hydrophobised cellulose-APP composites

In the previous report on cellulose-laponite film, we have confirmed that hydrophobic treatments had a significant effect on the cellulose samples, increasing the printing (conductive Ag-ink) resolution^46^. It is envisaged that it will show similar performance for this system. As a result, we did not repeat the Ag-ink printing studies with cellulose-APP composite.

### Biodegradability of cellulose-APP composites

**Figure 8:**
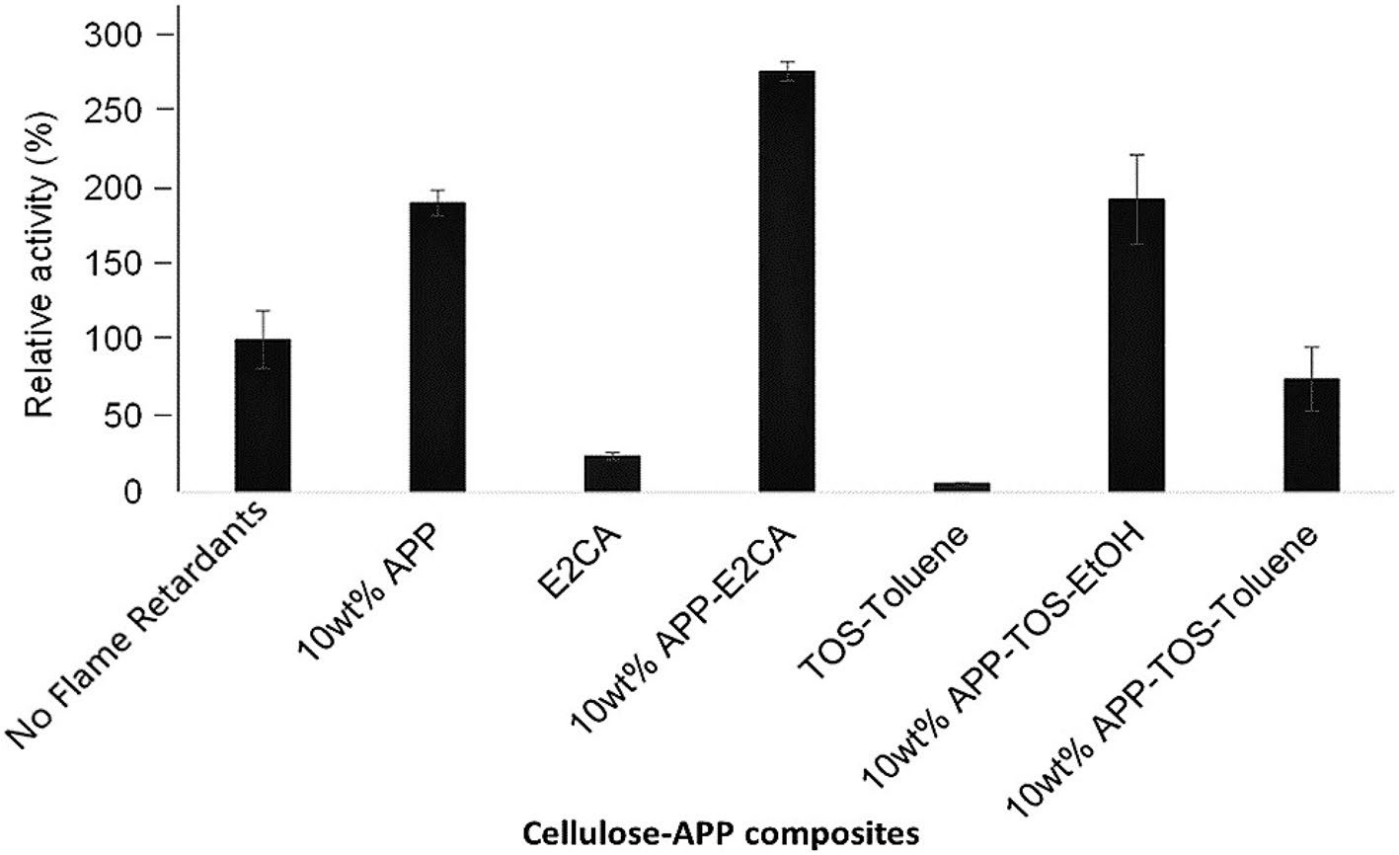
Cellulase activity on cellulose-APP composites.

When biodegradability studies were carried out with cellulases, APP had a positive effect on the enzymatic activity (Fig. 8). Cellulase activity increased by two-fold when APP was used as a flame retardant (in Fig. 8, no flame retardants and 10 wt% APP). It was also verified that APP is not acting as enzymatic activator when APP was tested as a powder (data not shown). Since APP is soluble in the reaction buffer (citrate pH 5), the hypothesis is that APP leaches out from cellulose film leaving more surface area available for cellulases than in its absence. ATR-FTIR confirmed that after incubating cellulose-APP composites with citrate buffer (without any enzyme), the intensity of characteristic peaks of APP were reduced (Fig. S-4). While composites were coated in absence of APP, the activity decreased (Fig. 8, E2CA and TOS-Toluene). Either the coating agents avoided the contact between cellulases and cellulose^46^ or cellulases get adsorbed onto the hydrophobic surface and affect their binding to cellulose^64–66^. The most surprising result was observed when cellulase activity measured on cellulose-APP composite hydrophobised with E2CA (Fig. 8, 10 wt% APP-E2CA) and the enzymatic activity increased by almost three times. Although the aim of this work is not to study substrate-enzyme interactions some physico-chemical reasons can be speculated for this increase. The polymeric form of hydrophobising agent E2CA (PECA) may forms a porous film on the surface of cellulose-APP composite, which may favous more interactions between cellulases and cellulose, leading to more enzymatic activity^49,67^. On the other hand, the differences in the enzymatic hydrolysis of TOS coated cellulose-APP composites (Fig. 8, 10 wt% APP-TOS-EtOH and 10 wt% APP-TOS-Toluene) confirms the effect of the solvent medium (ethanol or toluene) used during the hydrophobising process of composite with TOS. These findings demonstrate that the prepared cellulose-APP composite is biodegradable in nature irrespective of surface functionalisation.

## Conclusions

Cellulose composites were prepared with or without APP by a simple dissolution process. The EMIMAc was used as an efficient and recoverable dissolution medium without adding any organic solvent. The addition of APP into the cellulose increased the thermal and flame retardancy properties and proved that APP is an efficient flame retardant. The hydrophilicity of the cellulose-APP composite was reduced by treating it with hydrophobic agents by a simple dip-coating method; TOS was confirmed to be an efficient hydrophobic agent and toluene a suitable solvent medium to prepare the cellulose-APP composite with durable hydrophobic surface. Moreover, the prepared cellulose-APP composite was water resistant and the surface can be alterable for the printing process. Mechanical properties of the cellulose-APP composites were not altered whatever the surface treatment applied; more precisely the tensile elastic modulus of the films was not significantly decreased after surface modifications. The enzyme biodegradation studies on the cellulose-APP composites revealed that the coating with the hydrophobic agent substantially diminished the hydrolysis of cellulose by cellulases. However, the presence of APP in the composites enhanced the activity of cellulases, probably by increasing the surface area available for cellulases when APP leached out in the reaction media. Therefore, the prepared cellulose-APP composite materials may be suitable to use as a biodegradable PCB materials and this study paves the way for developing materials for advanced biodegradable PCBs and other electronic products.

## Supplementary Information

Supplementary Information.

## Data Availability

The corresponding author will provide the datasets generated for this study on request with valid reason.
