# Supplementary material for: Facile preparation of flame-retardant cellulose composite with biodegradable and water resistant properties for electronic device applications
Source: Sci Rep. 2023 Feb 23;13:3168. doi: 10.1038/s41598-023-30078-0 (PMC9950140; doi:10.1038/s41598-023-30078-0)
Supplement: Supplementary file 1 — Supplementary Information. [file 41598_2023_30078_MOESM1_ESM.docx]

**Supporting information**

**Facile preparation of flame-retardant cellulose composite with biodegradable and water resistant properties for electronic device applications**

**Saravanan Chandrasekaran,*^1, 4^ Alvaro Cruz-Izquierdo,^1^ Remi Castaing,^2^ Baljinder Kandola^3^ and Janet L Scott ^1^**

**^1^**Centre for Sustainable Chemical Technologies and Department of Chemistry,

University of Bath, Claverton Down, Bath, BA2 7AY, United Kingdom.

^2^Material and Chemical Characterisation Facility (MC²), University of Bath, Claverton Down, Bath, BA2 7AY, United Kingdom.

^3^Institute for Materials Research and Innovation, University of Bolton, Deane Road, Bolton, BL3 5AB, United Kingdom.

^4^ Department of Chemistry, School of Engineering, Presidency University, Rajanukunte, Itgalpura, Bangalore – 560064, India.

Email: [saravanan.chempoly@gmail.com](mailto:saravanan.chempoly@gmail.com); [saravanan@presidencyuniversity.in](mailto:saravanan@presidencyuniversity.in)

**TGA of cellulose-FRs composites:**

**Figure S-1:** TGA analysis on cellulose-FRs composites with 10 wt% of CaCO_3_ and Al_2_O_3_ under air at 5°C/min.

**TGA of cellulose-APP composites:**

**Figure S-2:** TGA curves of cellulose-APP composite before and after surface treatment with E2CA and TOS in ethanol or toluene under air at 5°C/min.

**Table S-1:** Effect of addition of flame-retardants on the limited oxygen index and flammability of the cellulose.

| **Cellulose composites** | **LOI (%)**  **(ASTM-D2863)** | **Flammability tests UL-94 HB, time to burn marked area of samples in secs (Std. dev)**  **(ASTM D 635-03)** | **Test Result**  **(pass = burning rate is less than 3"/min)** |
| --- | --- | --- | --- |
| 10wt% - Al_2_O_3_ | 19.6 | 27.0 (± 1.5) | Fail |
| 10wt% - CaCO^3^ | 20.4 | 27.4 (± 0.5) | Fail |

**
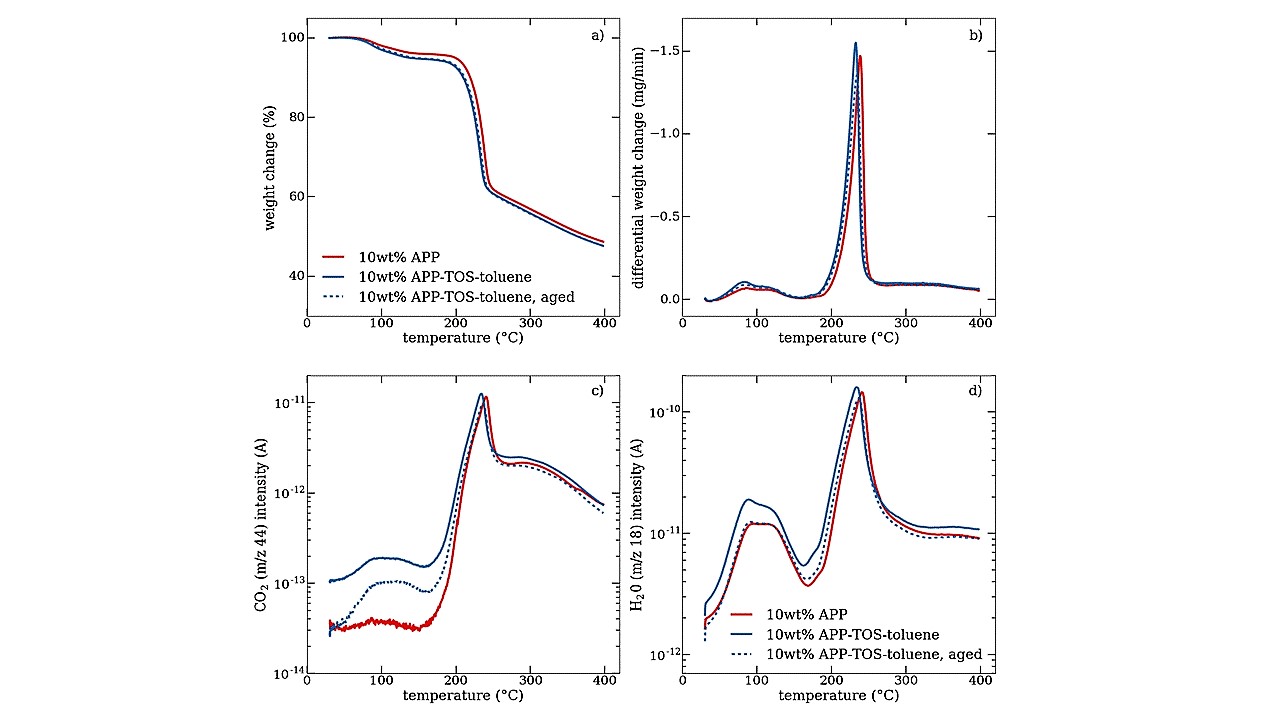
**

**Figure S-3:** Results of the thermogravimetric study of cellulose-APP composite under argon gas, depending on the surface treatment, a) weight changes versus temperature, b) differential weight changes versus temperature, c) mass spectrometer intensity of CO_2_ (m/z 44) versus temperature, and d) mass spectrometer intensity of H_2_O (m/z 18) versus temperature. The scanning rate was 5 *°C* /min.

Cellulose-APP composite was subjected to thermal degradation studies using thermogravimetry coupled with mass spectrometry under inert gas. Carbon dioxide and water are the main by-products during the pyrolysis of cellulose. Therefore, we simultaneously monitored the evolution of carbon dioxide and water vapour using mass spectrometry. The results are presented in Figure S-3 for a non-hydrophobised cellulose-APP composite, a composite treated with TOS in toluene and the corresponding aged (1-month) sample. All the three samples show the same features at very similar temperatures. Water vapour is released between 80 °C and 120 °C as the result of a small weight loss around 5 %. Then both carbon dioxide and water are released as the sample is showing a sharp mass loss at the temperatures at 239 °C, 233 °C and 234 °C, respectively in the three samples. Those temperatures are extracted from the differential weight profile (Fig S-3b). Between 150 °C and 400 °C, the material has lost 47 % of its initial mass. After compensating for the weight loss due to the evolved moisture at low temperatures, the char residues at 400 °C are 50.6 %, 50.2 % and 50.1 % for the non-hydrophobised cellulose-APP composite, the composite treated with TOS and the aged sample respectively. As a conclusion, the surface treatment shows no influence on the thermal degradation of cellulose with 10 wt% APP. Furthermore, it seems that there is no change in the material stability after 1 month of aging.

**ATR-FTIR of cellulose-APP composites:**

**
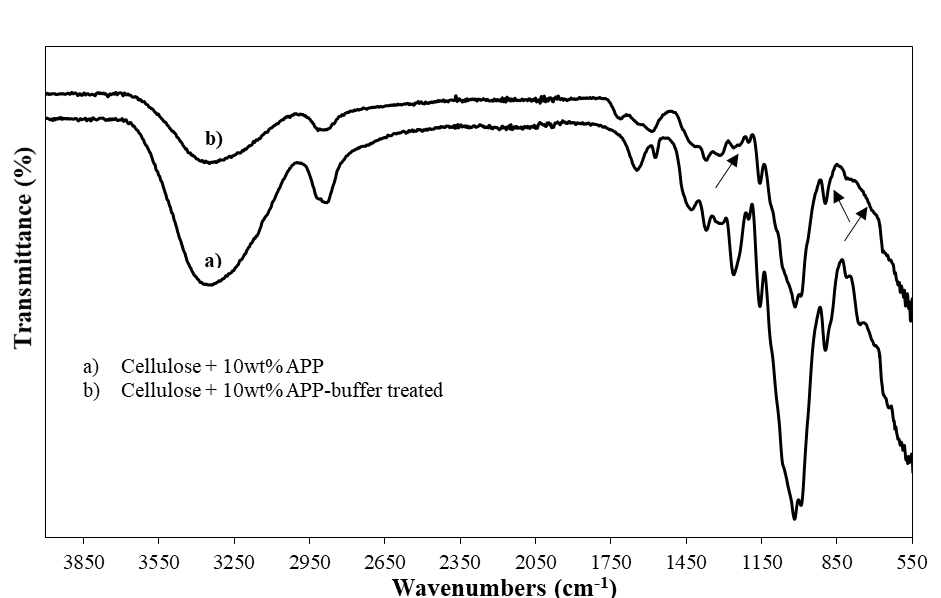
**

**Figure S-4:** ATR-FTIR of a) Cellulose-APP composite and b) Cellulose-APP composite -buffer treated.
